# Tumor circadian clock strength influences metastatic potential and predicts patient prognosis in luminal A breast cancer

**DOI:** 10.1073/pnas.2311854121

**Published:** 2024-02-06

**Authors:** Shi-Yang Li, Jan A. Hammarlund, Gang Wu, Jia-Wen Lian, Sacha J. Howell, Robert B. Clarke, Antony D. Adamson, Cátia F. Gonçalves, John B. Hogenesch, Ron C. Anafi, Qing-Jun Meng

**Affiliations:** ^a^Division of Cell Matrix Biology and Regenerative Medicine, School of Biological Sciences, Faculty of Biology, Medicine and Health, University of Manchester, Manchester M13 9PT, United Kingdom; ^b^School of Biomedical Engineering, Science and Health Systems, Bossone Research Center, Drexel University, Philadelphia, PA 19104; ^c^Division of Human Genetics, Center for Circadian Medicine, Department of Pediatrics, Cincinnati Children’s Hospital Medical Center, Cincinnati, OH 45229; ^d^Division of Immunobiology, Center for Circadian Medicine, Department of Pediatrics, Cincinnati Children’s Hospital Medical Center, Cincinnati, OH 45229; ^e^Breast Biology Group, Manchester Breast Centre, Division of Cancer Sciences, Faculty of Biology, Medicine and Health, University of Manchester, Manchester M20 4GJ, United Kingdom; ^f^Department of Medicine, Chronobiology and Sleep Institute, Perelman School of Medicine, University of Pennsylvania, Philadelphia, PA 19104

**Keywords:** breast cancer, circadian data ordering, circadian medicine, metastasis, prognosis

## Abstract

Studies in shift workers and model organisms link circadian disruption to breast cancer. However, molecular circadian rhythms in noncancerous and cancerous human breast tissues and their clinical relevance are largely unknown. We reconstructed rhythms informatically, integrating locally collected, time-stamped biopsies with public datasets. For noncancerous breast tissue, inflammatory, epithelial–mesenchymal transition (EMT), and estrogen responsiveness pathways show circadian modulation. Among tumors, clock correlation analysis demonstrates subtype-specific changes in circadian organization. Luminal A organoids and informatic ordering of luminal A samples exhibit continued, albeit dampened and reprogrammed rhythms. However, CYCLOPS magnitude, a measure of global rhythm strength, varied widely among luminal A samples. Cycling of EMT pathway genes was markedly increased in high-magnitude luminal A tumors. Surprisingly, patients with high-magnitude tumors had reduced 5-y survival. Correspondingly, 3D luminal A cultures show reduced invasion following molecular clock disruption. This study links subtype-specific circadian disruption in breast cancer to EMT, metastatic potential, and prognosis.

## Significance

Collecting time-course breast cancer biopsies is difficult. As a result, the influence of daily rhythms on breast tumor biology remains a mystery and physicians cannot personalize the timing of cancer therapies. We used machine learning to overcome this barrier, integrating data from hundreds of patients and ordering these data along circadian time. We identified rhythmic genes and pathways in normal human breast tissue and dampened and reprogrammed rhythms in luminal A breast cancers. Critically, patients with luminal A tumors that showed stronger global expression rhythms had an reduced 5-y survival. These same tumors showed increased cycling of EMT pathway genes. Using 3D cultures of patient-derived tumor cells, we show that luminal A clocks regulate cell invasion and metastasis.

Worldwide, breast cancer is the most common cancer among women (1–3). Over the last decades, the introduction of early detection programs, combined with improvements in systemic therapies, has reduced breast cancer mortality (2–4). Yet, resistance and subsequent relapse remain major issues (3). Women lose more disability-adjusted life years to breast cancer than to any other cancer (5). Adverse effects frequently compromise quality of life (3, 6–8). There remains a clear need to improve the therapeutic index for breast cancer treatments.

Recent research has highlighted the critical role of cell-intrinsic circadian rhythms in disease (including cancer) and medicine (9–12). The circadian (~24-hourly) clock is evolutionarily ancient and highly conserved, permitting cells to anticipate daily environmental changes through temporally coordinated metabolic and gene expression profiles (13–15). A series of transcription-translational feedback loops form the molecular circadian clock (13–16). The positive arm of the central loop includes the transcriptional activators *CLOCK* and *BMAL1* (13–16). The negative arm, which includes the Cryptochrome (*CRY1/CRY2*) and Period (*PER1/PER2/PER3*) genes, later represses translation (13–16).

Epidemiological and animal studies suggest that night shift work that disrupts circadian rhythms increases the risk of developing breast and other cancers, prompting the WHO to classify night shift work as a probable carcinogen (17–19). Acting in part through an immunosuppressive shift in the tumor environment, chronic circadian disruption increases mammary cancer cell dissemination and metastasis in a mouse model of tumorigenesis (20).

Beyond this basic biology, the molecular circadian clock regulates thousands of genes in a cell and tissue-specific manner. About half of top 100 best-selling drugs in the United States target the product of a circadian gene in mouse tissues (16). Decades of clinical experience show that time of day can influence chemotherapeutic activity (21, 22). However, the widespread translation of circadian biology in oncology remains slow and serendipitous (23). Mechanistic knowledge about the unique molecular rhythms in distinct tumors and normal human tissues must be improved. Repeated biopsies or time course sampling from large numbers of human patients is neither safe nor practical. As a result, clinically relevant molecular rhythms remain unknown, and opportunities for targeted circadian therapies unrealized. In addition, while some cancer models demonstrate a complete lack of rhythms, other models (like U2OS cells) show continued rhythms (24). Indeed, informatic analysis of intact hepatocellular carcinoma has shown disrupted yet persistent transcriptional rhythms (25).

To overcome this problem, we adopted a hybrid study design, optimizing a machine learning algorithm, CYClic Ordering by Periodic Structure (CYCLOPS), to integrate and order transcriptomic data from hundreds of patients (25–27) (*SI Appendix*, Fig. S1*A*). We combined deep sequencing of a small number of newly collected, time-stamped, paired clinical samples with large RNASeq datasets from the Tissue Cancer Genome Atlas (28) (TCGA) and the Genotype-Tissue Expression (29) (GTEx) project where the circadian time of sample collection is unknown.

## Results

### Profound Changes in Clock Gene Expression and Circadian Organization in Time-Recorded Breast Cancer Biopsies.

To assess and improve the accuracy of informatic predictions and to enable direct comparisons of transcriptional changes in circadian genes, we collected 43 pairs of fresh human breast samples (noncancerous and paired tumors from the same individuals) from patients undergoing mastectomy at the Nightingale Breast Centre, Manchester, United Kingdom (Patient demographics on *SI Appendix*, Table. S1). Noncancerous tissues were collected at least 4 cm away from tumors (*SI Appendix*, Fig. S1*A*). Clinical pathologists assessed the immunohistochemical expression of estrogen receptors (ER), progesterone receptors (PR), and human epidermal growth factor 2 receptors (HER2) in each tumor. Tumors were then classified as luminal A (ER+, PR−/PR+, HER2−), luminal B (ER+, PR−/PR+, HER2+), HER2 (ER−, PR−, HER2+), and TNBC (triple-negative breast cancer) (ER−, PR−, HER2−) based on receptor status (30). Tumor samples included luminal A (N = 29), luminal B (N = 3), HER2 (N = 2), and triple-negative breast cancers (TNBC, N = 9) (*SI Appendix*, Fig. S1*B*). Resection times of all samples were recorded (*SI Appendix*, Fig. S1*C*). To identify noncancerous and tumor areas we employed Hematoxylin and Eosin-Y staining (H&E staining) and immunohistochemistry using epithelial and stromal markers (Cytokeratin 8 and Vimentin, respectively) (*SI Appendix*, Fig. S1*D*). The normal breast contains organized acinar and lobular structures, whereas tumor regions lack regular glandular structures. We performed RNA sequencing following RNA isolation. Based on these RNAseq data, the expression of most clock genes is significantly altered in breast cancer tissues. Compared to the paired noncancerous samples, we observed significant downregulation of *PER1*, *PER2*, *CRY2*, *HLF*, *TEF*, and *NFIL3* (*SI Appendix*, Figs. S1*E* and S2). In contrast, *CLOCK*, *NPAS2*, *CIART/CHRONO*, *BHLHE40*, *RORA*, and *RORC* were significantly up-regulated in these breast tumors (*SI Appendix*, Figs. S1*E* and S2).

To examine core clock organization in these breast tumors, we performed Spearman’s correlation coefficient analysis (27, 31–33). We limited our analysis to paired breast tumors and non-cancerous samples with a sequencing depth > 20 million reads. The noncancerous breast tissues demonstrated core-clock correlation patterns mirroring those seen in mice (*SI Appendix*, Fig. S1*F*) (16). For example, in the noncancerous tissue, clock activators *BMAL1(ARNTL)* and *NPAS2* reach peak expression at a similar time. Samples with high *BMAL1* expression have high *NPAS2* expression, and the expression of *BMAL1* and *NPAS2* are positively correlated (indicated as red in *SI Appendix*, Fig. S1*F*). On the other hand, CHRONO(CIART) peaks when *BMAL1* is near its trough and the expression of the two genes is negatively correlated (indicated as blue in *SI Appendix*, Fig. S1*F*). The strong similarity between the clock correlation patterns seen in the noncancerous tissue (*SI Appendix*, Fig. S1*F*, Zstat score of 20.71) and the mouse model suggests a functional clock network. In contrast, a weaker overall correlation in breast tumors (*SI Appendix*, Fig. S1*F*, Zstat score of 9.28) suggests a weakening of core circadian organization in these samples.

### Transcriptional Circadian Rhythms Are Evolutionarily Conserved in Noncancerous Human Breast and Mouse Mammary Tissues.

We adopted a hybrid study design (27) to evaluate circadian time in human noncancerous breast tissues. We used informatic tools to integrate RNAseq data from newly collected time-stamped breast samples (N = 26, with noncancerous samples with >20 million reads) with RNAseq data from female breast samples in public databases. We incorporated data from TCGA (28) and GTEx (29) (*SI Appendix*, Table S2). We did not include samples collected in centers where only a small number (n < 5) of noncancerous samples were processed.

Differences between sample collection sites, processing methods, and patient populations complicate the use of aggregate data. These differences may be particularly problematic when different centers have different biases in collection time. We modified the CYCLOPS (25) neural network to accommodate explicit confounding variables, simultaneously learning confounder adjustments and a common circular structure that explains the variance of the combined data: CYCLOPS 2.0 (*SI Appendix*, Fig. S3*A*). We benchmarked CYCLOPS 2.0 on actual, semisynthetic, and fully synthetic data with different temporal biases (*SI Appendix*, Fig. S3 *B–D*). CYCLOPS 2.0 demonstrates improved accuracy with realistic levels of noncircadian noise. Finally, we allowed the ordering process to use information from a subset of time-stamped samples. We performed 10-fold cross-validation to determine the relative weight given to predicting time in these samples and identify a common circular structure for all samples.

**Fig. 1.**
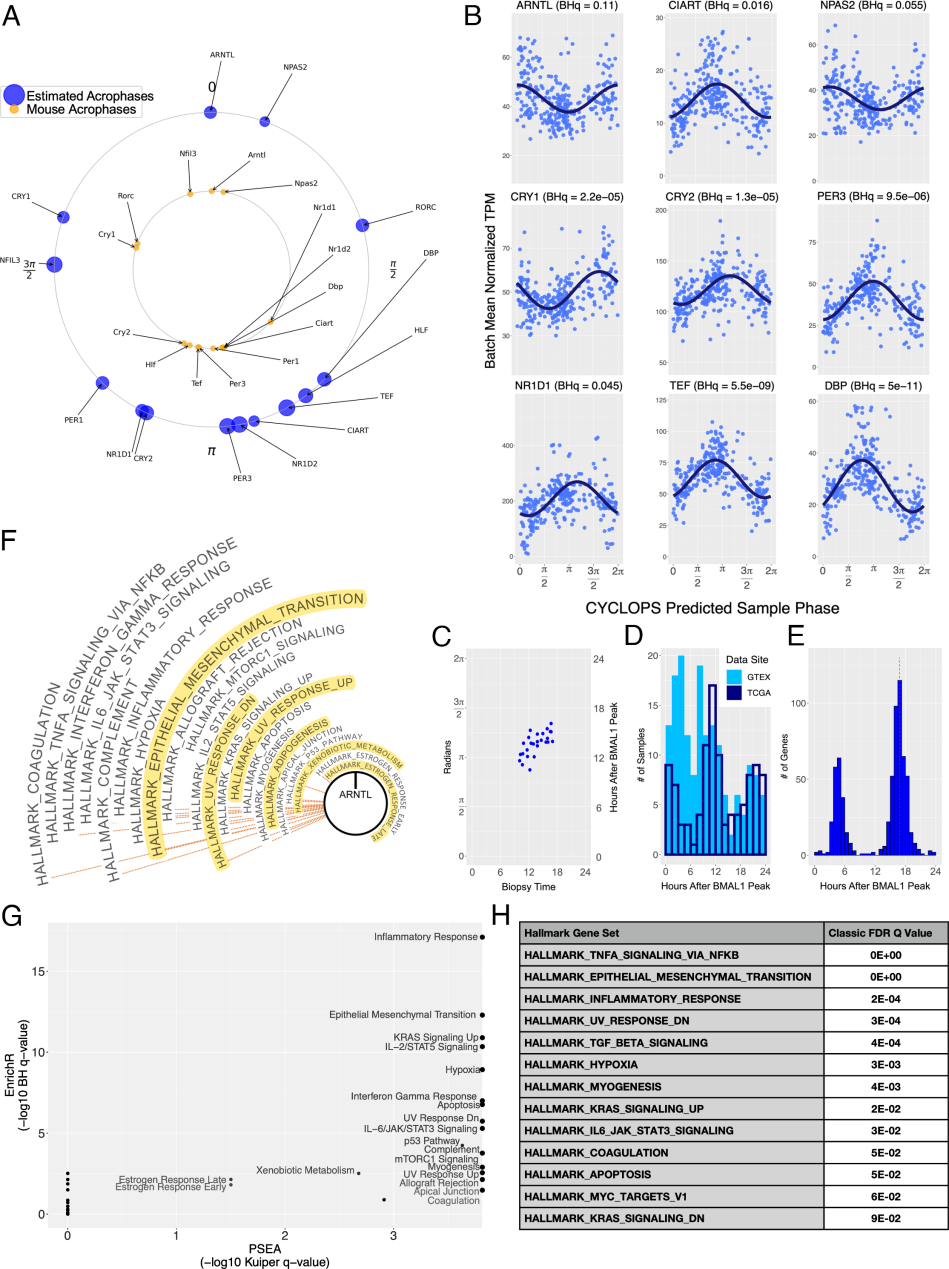
CYCLOPS reconstructed rhythms in noncancerous human breast samples. CYCLOPS was used to estimate sample circadian phase in noncancerous human breast tissue, including samples newly collected in Manchester, United Kingdom (N = 26), from GTEx (N = 167), and TCGA (N = 106). (*A*) Inferred time of peak transcript expression phase (acrophase) for select core clock genes in noncancerous human breast (outer, blue). The mouse paralogue acrophases (averaged across mouse tissues) are shown in the inner circle (inner, orange). In the mouse, time 0 was defined by the peak time of *Arntl* (*Bmal1*) expression. Rhythmicity was assessed by modified cosinor regression. All transcripts shown in blue had a regression *P* < 0.05. The human ordering was aligned to match the mouse acrophases. (*B*) Transcript expression was fit using cosinor regression including batch collection site. Batch-adjusted transcript expression is plotted as a function of CYCLOPS-predicted sample phase. The best-fit sinusoid and its significance are shown for each transcript. Solid lines represent BHq < 0.05. (*C*) Optimal alignment of CYCLOPS-predicted sample phases for the subset of time-stamped samples collected in Manchester (${\text{Corr}}_{\text{Fisher}}=0.69,\;p_{\text{Fisher}}<0.01$ ). (*D*) Histogram of inferred sample collection hour for noncancerous GTEx (filled) and TCGA (outlined) data. Sample phases are aligned as in (*A*). (*E*) Histogram of transcript acrophases including all significantly cycling transcripts in noncancerous breast tissues (BHq < 0.05). (*F*) PSEA was applied to CYCLOPS-ordered noncancerous human breast data. MSigDB hallmark gene sets with BHq < 0.05 are shown. Gene sets graphed furthest from the center had the most significant phase coordination. Gene sets highlighted in yellow also demonstrated phase coordination in mouse mammary tissues. (*G*) EnrichR was used to identify MSigDB Hallmark gene sets overrepresented among cycling genes in human noncancerous breast (BHq < 0.05, relative amplitude >0.33). The significance of pathway overrepresentation (−log BHq) is plotted against the significance of pathway phase coordination identified by PSEA (−log BHq). (*H*) Transcripts were ranked by cosinor regression F statistic. GSEA was applied to the ranked list. Hallmark gene sets with BHq < 0.1 are shown.

We identified the human orthologues of transcripts that cycle in mouse mammary tissue (34). Combining these with the human orthologues of transcripts that cycled in >75% of mouse tissues (16), we constructed a circadian “seed gene” list appropriate for ordering human breast tissue. Ordering the combined dataset using these seed genes and including temporal information from the 26 time-stamped human samples, the CYCLOPS smoothness and ordering metrics for the entire dataset meet previously established standards (Statsmooth = 0.75; Staterror = 0.015). After ordering, we used modified Cosinor regression (25, 35) to identify cycling transcripts and estimate their amplitude and acrophase (peak expression time). With the notable exception of *RORC*, the relative acrophases of core-clock transcripts reconstructed from noncancerous human breast tissues are in good accord with the well-established ordering of these transcripts in other mouse and human tissues (Fig. 1 *A* and *B*). The CYCLOPS-predicted sample phases show a significant correlation with the known sample collection times of the 26 subjects (Corrcirc = 0.7, *P* < 0.005) (Fig. 1*C*). As expected, the sample phases assigned to the TCGA biopsies were concentrated during the presumed working day (Fig. 1*D*). In contrast, phases assigned to the autopsy-derived GTEx samples was more uniformly distributed, but still showed a significant bias (Fig. 1*D*). The expanded Cosinor model explicitly accounted for differences in expression due to sequencing sites or source databases. At a BHq threshold of 0.05, we identified ~2,000 genes as rhythmic. Requiring a relative cycling amplitude [amplitude/MESOR (Midline Estimating Statistic of Rhythm)] greater than 1/3 as a measure of likely biological significance, reduced the number of identified cycling transcripts to ~650 (Dataset S1). As observed in other tissues, there are clear circadian “rush hours” where many rhythmic transcripts peaked (Fig. 1*E*) (16, 26, 36).

To put these results in a broader biological context, we used phase set enrichment (PSEA) (37) to identify annotated gene sets and biological pathways where the constituent cycling transcripts exhibited circadian concentration and were not uniformly distributed across the circadian day. As in the mouse, pathways related to adipogenesis, EMT, and estrogen responsiveness, show circadian orchestration (Fig. 1*F*). Using both gene set enrichment (38) and overrepresentation approaches (39–41), we also labeled pathways that were enriched for cycling genes. In addition to the abovementioned pathways, various immune and cell cycle pathways show marked circadian orchestration (Fig. 1 *G* and *H*).

### ER Activity Correlates with Circadian Organization and Function in Breast Cancer Subtypes.

**Fig. 2.**
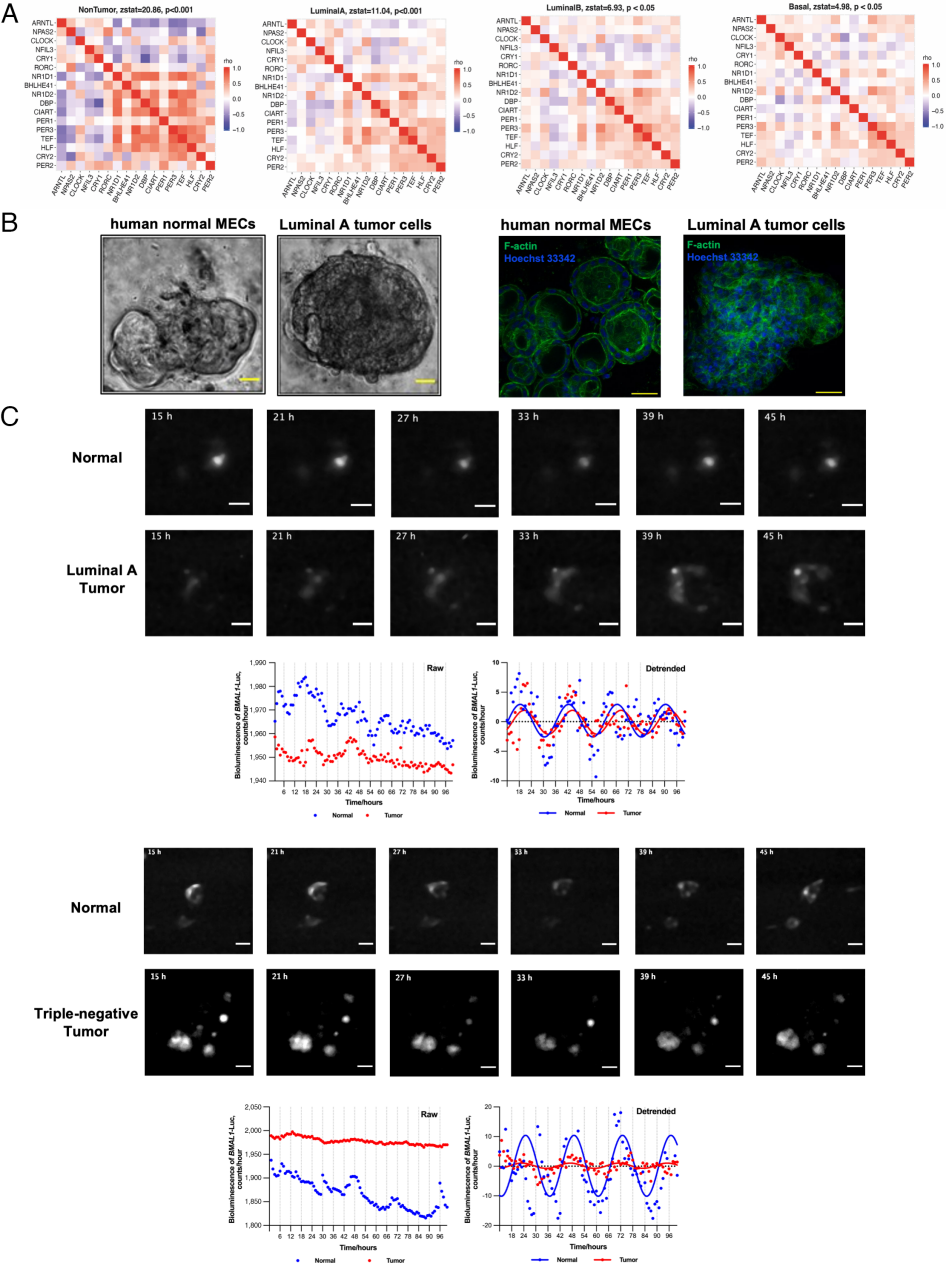
Subtype-specific circadian rhythm dysfunction in breast cancers. (*A*) Evaluation of core circadian organization in human breast cancer subtypes from TCGA data. Heatmaps depicting the Spearman correlation between selected core clock genes are shown for noncancerous breast tissue (N = 111), luminal A (N = 532), luminal B (N = 203), and basal/TNBC (N = 181). The zstat value and *P*-value are computed using a Mantel test and a reference correlation matrix of clock and clock-associated genes from the mouse atlas data. Higher Zstat scores denote a stronger resemblance to the established reference for healthy tissues. (*B*) *Left*, Representative images of mammary organoids derived from noncancerous breast and paired tumor tissues from the same individual. (Scale bar: 50 µm.) *Right*, Immunostaining demonstrates morphological structures. Hoechst 33342 was used for the nucleus and F-Actin was used for the cytoskeleton. (Scale bar: 50 µm.) (*C*) Circadian rhythms in matched normal and tumor organoids were monitored by a LV200 system. Representative bioluminescence images of organoids transduced with *BMAL1*-Luc from luminal A (N = 4) and TNBC (N = 3) subtypes were taken at 6-h intervals. (Scale bar: 100 µm.) Both raw and detrended signals are shown (red traces, tumor organoids; blue traces, nontumor organoids).

Our analysis of locally collected samples combined data from biologically distinct breast tumor types. We applied clock gene correlation analysis (27, 31–33) to TCGA breast tumor data to evaluate core clock organization in cancer subtypes. The expression of the PAM50 panel genes was used to define cancer subtypes (42, 43). As with the time-stamped samples, noncancerous breast tissues from TCGA show core clock organization that closely mirrors the established healthy consensus with a Zstat score of 20.86. The luminal A samples demonstrate weaker but still considerable evidence of intact core clock organization with a Zstat score of 11.04. On the other hand, luminal B and basal/TNBC samples exhibit disrupted correlation patterns with Zstat scores of 6.93 and 4.98, respectively (Fig. 2*A*). Using a permutation test (32, 33), we directly compared the Zstat scores of these three tumor subtypes. The correlation patterns in luminal A tumors were significantly stronger than those seen in luminal B tumors (*P* = <0.01), while the Zstat scores of the luminal B and basal groups were not significantly different. The relatively small number of HER2 samples in the TCGA database prevented evaluation of core clock organization in this tumor type.

We hypothesized that circadian function, like core clock organization, varies among breast cancer subtypes. To confirm these predictions, we assessed circadian rhythms in vitro, using cancerous and noncancerous breast tissues from the same individuals. We derived organoids from primary epithelial cells. This model more closely mimics in vivo physiological functions of mammary epithelia (44–46). The organoid cultures from normal breast tissues showed typical acinar structures. In contrast, breast tumor organoids showed disrupted cell polarity (Fig. 2*B*). After lentiviral transduction of a *BMAL1*-Luc circadian reporter, we imaged bioluminescence signals using an LV200 imaging system (Olympus). Noncancerous organoids showed robust circadian rhythms. All four patient-derived luminal A tumor organoids tested showed persistent (*P* < 0.01) but weakened rhythms (Fig. 2*C*, Movie S1, N = 4). However, we did not observe consistent *BMAL1*-Luc rhythms in TNBC tumor organoids. Only one of three samples showed significant rhythms (Fig. 2*C*, Movie S2, N = 3). Using either a *BMAL1*-Luc reporter or time-course western-blot of clock factors, we also observed variable clock function among established breast cancer cell lines representing various tumor subtypes (*SI Appendix*, Fig. S4 *A–C*). The MCF-7 cell line (representative of ER+ luminal A) exhibits circadian rhythms, while the ER-negative MDA-MB-231 and SKBR3 cell lines do not (*SI Appendix*, Fig. S4 *B* and *C*). In unsynchronized cells, we observed altered average expression levels of core clock genes among the three cell lines (*SI Appendix*, Fig. S4*D*).

As ER status is a key factor differentiating these tumor subtypes, we hypothesized that ER responsiveness is linked to breast cancer clock function. Indeed, stratifying breast tumor samples based on ER status indicates a strong correlation between ER expression and clock functionality (*SI Appendix*, Fig. S5*A*). To more directly determine whether ER signaling regulates circadian rhythms in breast cancer cells, *ERα* was knocked out of *BMAL1*-Luc MCF-7 cells using CRISPR-Cas9. We isolated single-cell colonies following cotransfection of sgRNA and Cas9 protein. We confirmed successful knockout by DNA sequencing, supported by the absence of *ERα* mRNA and protein (*SI Appendix*, Fig. S5 *B* and *C*). *ERα*-KO disrupted the expression of clock factors in MCF-7 cells compared to the control (*SI Appendix*, Fig. S5 *D* and *E*). In contrast to the robust 24-h rhythms in control MCF-7 cells, there was a complete loss of circadian *BMAL1*-Luc rhythms in all four clones of MCF-7 cells with *ERα-*KO (*SI Appendix*, Fig. S6*A*). 17β-Estradiol (E2), an estrogen hormone activating both ERα and ERβ, synchronized circadian rhythms in MCF-7 cells (*SI Appendix*, Fig. S6*B*). The ERα selective agonist PPT (Propyl Pyrazole Triol) also synchronized circadian clocks in MCF-7 cells in a dose-dependent manner (*SI Appendix*, Fig. S6*C*).

### CYCLOPS 2.0 Analysis Revealed Global Changes in Rhythmic Gene Expression Patterns and Pathways in Luminal A Samples.

**Fig. 3.**
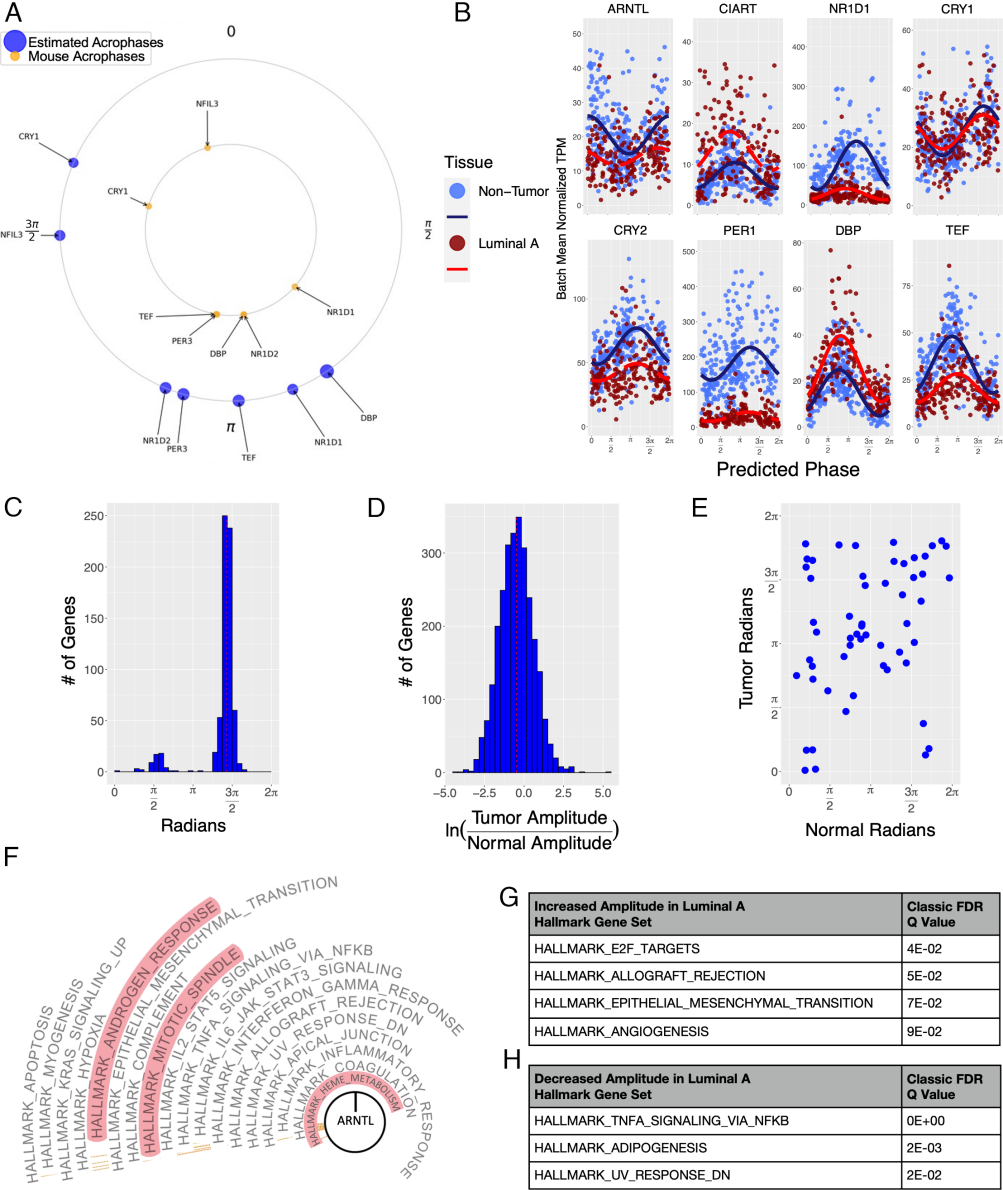
CYCLOPS reconstructed circadian rhythms from luminal A samples. CYCLOPS was used to estimate sample circadian phase in luminal A samples (N = 18 from Manchester and N = 193 from TCGA). (*A*) Acrophases are plotted for select core clock genes in luminal A tumors (outer, blue) and mouse paralogues (inner, orange). All transcripts in blue had a regression *P* < 0.05. The human ordering was aligned to match the mouse acrophases. (*B*) Transcript expression in luminal A samples was fit by cosinor regression including batch collection site. Separate models were fit to luminal A (red) and noncancerous samples (blue). Batch-adjusted transcript expression is plotted as a function of the CYCLOPS-predicted sample phase. Solid lines represent BHq < 0.05, and dashed lines represent a nonsignificant fit. (*C*) Histogram of luminal A transcript acrophases cycling with BHq < 0.05. (*D*) Histogram of the log amplitude ratio comparing luminal A and noncancerous genes, including transcripts that cycled in either luminal A or noncancerous samples with BHq < 0.05. (*E*) The estimated phase of luminal A tumor samples is plotted against the estimated phase of the matched noncancerous samples. (*F*) PSEA was applied to CYCLOPS-ordered luminal A data. Gene sets furthest from the center had the most significant phase coordination. All gene sets other than those highlighted in red also demonstrated phase coordination in noncancerous breast samples. (*G* and *H*) MSigDB Hallmark gene sets enriched for increased (*G*) and decreased (*H*) cycling in luminal A samples are shown. Transcripts significantly cycling in either luminal A or noncancerous samples (BHq < 0.05) were ranked by the log fold change in amplitude and analyzed by GSEA.

Guided by the evidence for persistent rhythms in luminal A tumors and the abundance of luminal A samples in the TCGA database, we next used CYCLOPS 2.0 to order luminal A tumors (*SI Appendix*, Table S3). There is likely significant noncircadian heterogeneity among luminal A samples. We projected the luminal A data onto the eigengene space computed from the noncancerous samples (25) to emphasize circadian variation. Using the CYCLOPS 2.0 model, we included data from both noncancerous and luminal A samples, now listing tumor status as a covariate. After ordering and applying cosinor regression to the luminal A samples, seven core clock genes, including *DBP*, *NR1D1*, *NR1D2*, *TEF*, *PER3*, *NFIL3*, and *CRY1*, meet the initial criteria for cycling (Fig. 3 *A* and *B*). At a BHq threshold of 0.05, we identified ~1,100 genes as rhythmic. Requiring a relative cycling amplitude greater than 1/3, we reduced the number of identified cycling transcripts to ~675 (Dataset S2). Of course, differences in sample size and noncircadian variability may have contributed to these changes. Thus, we used nested regression models as we (25) and others (47, 48) have done previously, to directly test for changes in cycling between luminal A and noncancerous samples (Dataset S3). This nested modeling approach tests the importance of tumor-dependent cycling parameters while accounting for tumor-dependent differences in mean expression. Luminal A samples showed significant differences in both core clock and clock output rhythms (Fig. 3 *A* and *B*). For example, while *TEF* shows decreased amplitude in the luminal A samples, its partner and structural/functional paralogue (49), *DBP*, shows increased amplitude. As in the noncancerous samples, luminal A samples show rush hours of rhythmic transcription (Fig. 3*C*). However, the proportion of samples assigned to the window that precedes the *ARNTL* (*BMAL1*) acrophase (inferred early evening) is much higher. Among transcripts that cycle in either luminal A or noncancerous samples, more transcripts lose as opposed to gain amplitude in luminal A samples (Fig. 3*D*). Of note, TCGA includes a limited number of matched luminal A tumors and noncancerous samples from the same patient. The sample phases assigned to the tumors and their noncancerous matches are poorly correlated (Fig. 3*E*).

At a gene set level, many pathways demonstrate continued circadian orchestration in the luminal A samples (Fig. 3*F*). PSEA reveals cycling in EMT, androgen responsiveness, immune, and inflammatory pathways. To identify pathways with enhanced rhythmicity in luminal A, we first ranked the full complement of genes that cycled in either luminal A or noncancerous tissue by the log fold change in amplitude. We then used GSEA to identify gene sets enriched for more marked amplitude increases in this ranked list (Fig. 3*G*). EMT and angiogenesis pathways, critical to cell invasion and growth support, show increased cycling in luminal A samples. Adipogenesis and pathways related to NFƙB signaling show reduced cycling using the same analysis (Fig. 3*H*).

### CYCLOPS Magnitude As a Measure of Global Circadian Rhythm Strength.

**Fig. 4.**
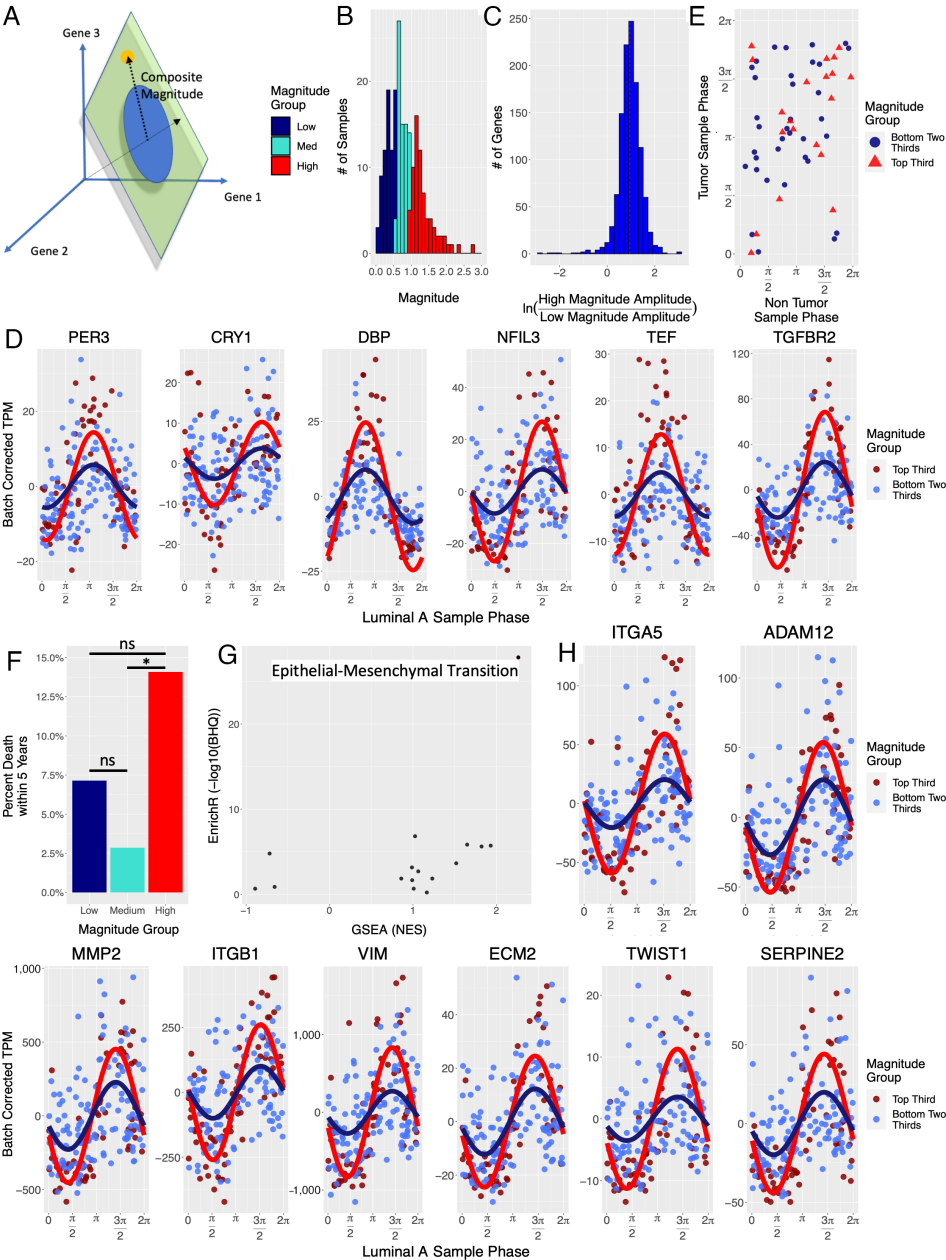
CYCLOPS clock magnitude varies in luminal A samples, correlates with EMT, and predicts prognosis. (*A*) Cartoon depicting CYCLOPS sample magnitude. CYLOPS projects the eigengene expression of each sample to a plane where the circular data structure is apparent. The angular position of any sample in the circle reflects its circadian phase. The radial distance from the center is the sample’s CYCLOPS magnitude (dotted line). This distance is a weighted sum of the amplitudes of individually cycling seed genes. (*B*) Histogram of luminal A CYCLOPS sample magnitudes colored by tertiles (N = 70). The distribution shows a long tail. (*C*) A histogram of transcript amplitude ratios comparing samples from the top-third and bottom-third magnitude groups. Transcripts that showed differential cycling by the CYCLOPS magnitude group were identified (BHq < 0.05). For differentially cycling transcripts, the cycling amplitude in the highest tertile was compared to the amplitude in the lowest tertile. (*D*) Batch normalized expression data are shown for representative core clock transcripts that exhibit differential cycling between magnitude groups (BHq < 0.05). Luminal A samples in the top tertile of CYCLOPS magnitude are shown in red and the bottom two tertiles in blue. The best-fit sinusoid is superimposed for each group. (*E*) The CYCLOPS estimated phase of luminal A tumor samples is plotted against the phase of matched noncancerous samples in TCGA. Samples are colored by the CYCLOPS magnitude group, distinguishing the top tertile from the bottom two tertiles. (*F*) Five-year patient mortality grouped by the tumor CYCLOPS magnitude group. Patient outcome data were obtained from the TCGA database. For each tertile/magnitude group, the percentage of patients who died within 5-y of diagnosis is shown. The increased risk in the high-magnitude tumor group remained statistically significant when evaluated in a logistic regression model that included patient age and the presence of known metastasis at diagnosis (*P* < 0.05). (*G*) Pathway overrepresentation (EnrichR) and enrichment analysis (GSEA) for transcripts that showed differential cycling (BHq < 0.05) among luminal A magnitude groups. The −log(BHq) from the EnrichR analysis is plotted against the normalized enrichment score (NES) from GSEA. (*H*) Batch normalized expression data for select transcripts in the epithelial–mesenchymal transition pathway. Data for the top tertile of CYCLOPS magnitude (red) and bottom two tertiles (blue) were fit to sinusoids.

While the amplitude of a single rhythmic waveform is well defined, meaningful, global measures of transcriptional rhythm strength still need to be established. For example, it is generally unknown whether high-amplitude circadian expression in some genes predicts high-amplitude circadian expression of others. CYCLOPS operates on eigengenes, i.e., global descriptors of expression that summarize the behavior of many cycling genes. CYCLOPS projects these data onto a plane where a circular structure is apparent. We use the angular position of any sample on this circle to infer its internal molecular phase. We also calculate the radial position of each sample. Geometrically, we interpret CYCLOPS magnitude (CMag) (Fig. 4*A*) as a weighted sum of the amplitudes of the individually cycling seed genes. This concept resembles the PCA plots of cycling gene expression by Brooks et al. (50). The distribution of CYCLOPS magnitudes obtained from the luminal A samples is broad with a long tail (Fig. 4*B*). Dividing samples into equal thirds based on CMag, we find that across all cycling transcripts, the amplitude of cycling is generally greater in high magnitude samples as compared to low magnitude samples (Fig. 4 *C* and *D*). Unlike luminal A samples as a whole (Fig. 3*E*), the circadian molecular phases assigned to high CMag luminal A samples generally match the phases assigned to their noncancerous pair (Fig. 4*E*). This suggests that higher CMag in luminal A samples is indicative of a more robust clock.

### Circadian Rhythm Strength Predicts Prognosis and Modulates Metastatic Potential.

We investigated whether rhythm strength influenced tumor biology and prognosis. Patients were stratified into thirds based on tumor CMag (low, medium, and high CMag). Using TCGA outcome data, we evaluated these patients’ 5-y survival. The risk of death was increased among patients with high-magnitude luminal A tumors (Fig. 4*F*). This difference is statistically significant (*P* = 0.047, ANOVA). The increased risk in the high-magnitude tumor group remains statistically significant when we tested its influence in a generalized (logistic regression) model that also included patient age and the presence of known metastases at diagnosis (*P* < 0.05). A high-magnitude tumor increased the relative risk ~1.5-fold, over and above the risk established by the other covariates. The predictive value of tumor magnitude remains significant in a model that includes the expression of *MKI67* (51, 52), the gene encoding Ki-67 protein, a proliferation marker known to have prognostic significance (*P* < 0.01). Of some note, while the same trend appears examining a broader outcome of death OR new cancer event (*SI Appendix*, Fig. S7), this trend is not statistically significant.

CMag is broadly associated with the cycling amplitude of many genes. Given its prognostic importance, we next identified rhythmic pathways that showed the most marked differences in high-magnitude samples. For each transcript that showed statistically significant cycling in luminal A samples, we compared the amplitude estimated from the high-magnitude samples (top third) to the amplitude estimated from lower-magnitude samples (bottom two-thirds). We leveraged both enrichment and overrepresentation approaches to analyze these results at the pathway level. When we compare high- and lower-magnitude samples, our analyses show that EMT-related genes exhibit the most pronounced changes in cycling (Fig. 4 *G* and *H*). Given the well-established role of EMT in tumor metastasis, we hypothesized that high-amplitude luminal A rhythms might modulate cell behavior and the potential for invasion.

**Fig. 5.**
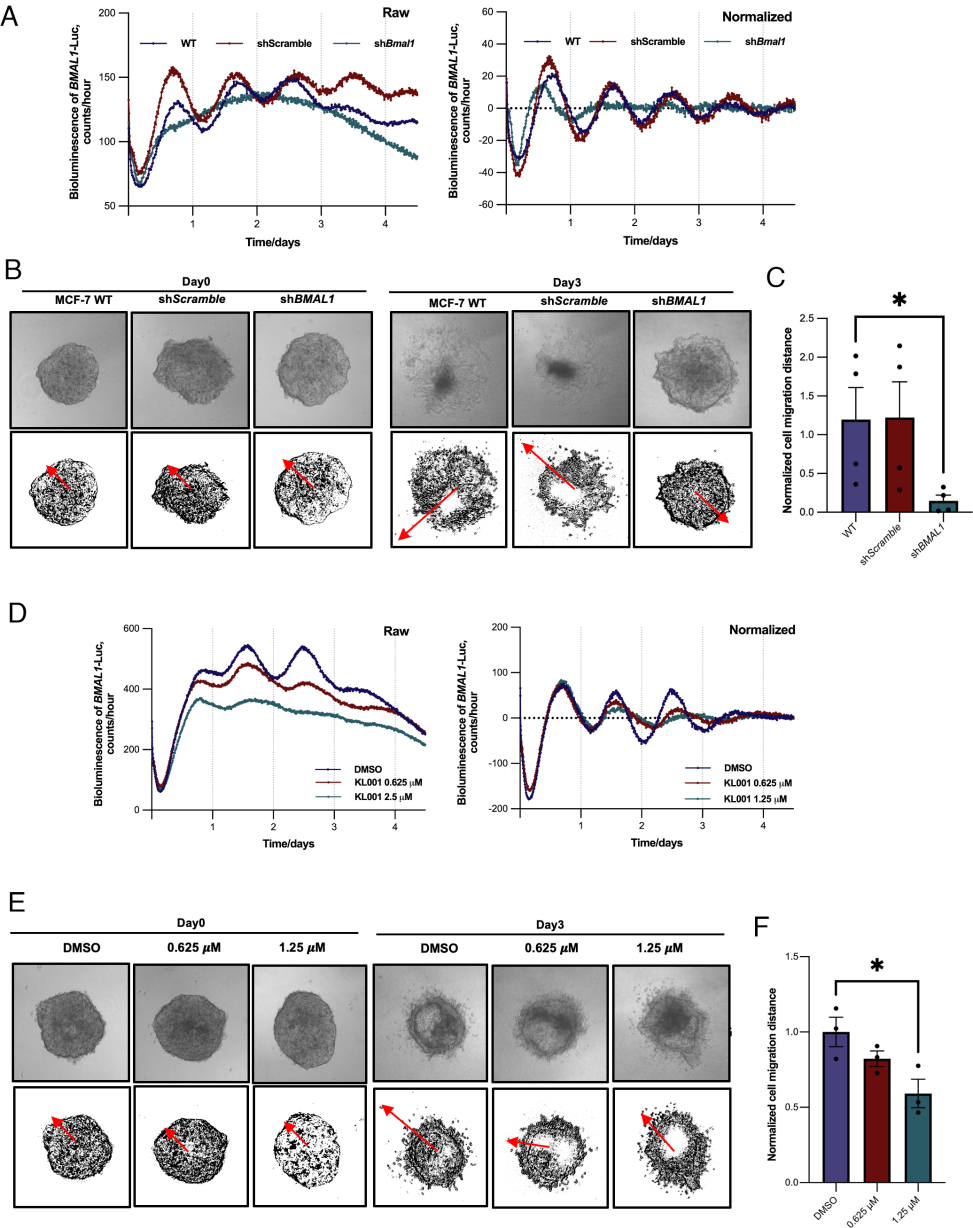
Invasion of MCF-7 cells into the 3D collagen matrix is suppressed by circadian clock disruption. (*A*) Representative traces of circadian rhythms in *BMAL1*-Luc expression in MCF-7 cells transduced with lentiviral sh*Scramble* or sh*BMAL1*. *Left*, raw data; right, normalized data. N = 3. (*B*) Hanging drop cell invasion assay of MCF-7 cells in a 3D collagen matrix. Representative images of cell migration are displayed at day 0 (*Left*) and day 3 (*Right*). (Scale bar: 100 μm.) N = 4. (*C*) The relative distance of cell migration was quantified and plotted. Data were normalized to WT which was set as 1. Unpaired *t* test, *P < 0.05, N = 4. (*D*) Representative traces of circadian rhythms in *BMAL1*-Luc expression in MCF-7 cells treated with KL001. N = 3. (*E*) Representative images of cell invasion assay of MCF-7 cells treated with KL001. (Scale bar: 100 μm.) N = 3. (*F*) Quantification of cell migration distance in (*E*), unpaired *t* test, *P < 0.05, N = 3.

To establish the role of circadian clocks in regulating breast cancer cell behavior, we used lentiviral shRNA for *BMAL1*, the essential clock factor, to disrupt cellular clock functions in rhythmic MCF-7 cells. As expected, the lack of *BMAL1* abolished circadian reporter rhythms in MCF-7 cells (Fig. 5*A*). We used hanging drop and cell invasion assays to evaluate the invasion of MCF-7 cells through a 3D collagen I matrix microenvironment. Circadian disruption through *BMAL1* deficiency inhibited the rate of cell invasion in both MCF-7 cells (*P* < 0.001, Fig. 5 *B* and *C*) and primary luminal A breast tumor cells (*SI Appendix*, Fig. S8*A*). Disrupting the molecular clock with the CRY1/CRY2 stabilizer KL001 also suppressed invasiveness (Fig. 5 *D–F*). MCF-7 cell proliferation was assessed by expression of Ki-67 and real-time quantitative IncuCyte imaging. *BMAL1* knockdown increased Ki-67 abundance (*SI Appendix*, Fig. S8*B*) and cell proliferation (*P* < 0.0001) (*SI Appendix*, Fig. S8*C* and Movie S3). As such, the loss of molecular clock rhythm in MCF-7 cells compromises breast cancer cell invasion into the 3D matrix, despite increasing cell proliferation.

## Discussion

This work used informatic ordering (CYCLOPS 2.0) to reconstruct temporal rhythms in noncancerous breast tissues and luminal A breast tumors, using data from both newly collected, time-stamped paired breast biopsies and public datasets. Our approach reveals cycling of inflammatory, EMT, and estrogen response pathway genes. Experiments with luminal A organoids show continued, albeit dampened rhythms. Disrupted rhythms are also evident in our informatic circadian reconstruction of luminal A tumors. Strikingly, retrospective analysis shows that luminal A cancer patients with high-rhythm-strength tumors had reduced 5-y survival. EMT pathway genes show the most marked increase in cycling when comparing tumors with higher and lower rhythm strength. 3D culture experiments using established luminal A cancer cell lines and primary luminal A cells show reduced invasion following molecular clock disruption. As such, our study links subtype-specific circadian disruption in breast cancer to EMT, metastatic potential, and prognosis.

A bidirectional web of transcription factors and direct protein–protein interactions couples the cell-intrinsic circadian clocks and the cell cycle. Core circadian clock genes include the likely tumor suppressors *PER1* and *PER2* (53–55). More recently, researchers have shown that the master clock activators *BMAL1* and *CLOCK* have anti-apoptotic roles, promoting liver cell proliferation through the cell cycle regulator *Wee-1* (56). On the other hand, oncogenes such as *c-MYC* or *KRAS* interfere with circadian pacemaking (57, 58).

The circadian-cancer connection may be vital in breast cancer. Several epidemiologic studies have now linked night shift work with breast cancer risk (17–19). In mouse models bearing primary mammary tumors or breast cancer xenografts, the efficacies of Doxorubicin and Celecoxib are time of day dependent (59, 60). However, the difficulty of obtaining time-course clinical samples across multiple circadian cycles in a large, clinically informative cohort hinders our understanding of circadian biology and its translation in human breast cancer. The application of circadian ordering methods and the hybrid informatic and clinical experimental study designs can be key tools in overcoming this obstacle.

Several supervised learning algorithms (e.g., BodyTime, TimeSignature, TimeTable, and ZeitZeiger) predict internal clock time from time-unknown human samples (61–64). These approaches require “training data” that spans the tissues and conditions covered in later applications. As a result, they are not designed for application to new tissues or disease states (such as solid tumors) without specific training data. In contrast, CYCLOPS (25) uses global descriptors of expression structure and unsupervised machine learning that identifies more general signatures of rhythmic processes. CYCLOPS requires a list of “seed genes” likely to cycle in a given tissue and assumes a fixed relative phase relationship between the cycling seed genes across subjects (e.g., *BMAL1* precedes *NR1D1* by a relatively fixed amount in the oscillations in each sample). More recently, Talamanca et al. (36) aimed to increase the power of this approach, focusing on GTEx data, where many sample tissues were taken from the same individuals. Their approach assumes that tissues obtained from the same individual at the same time are at the same molecular phase.

In this study, the limitations of having a single tissue type are compounded by the need to aggregate data from several sources. We specifically tailored our modifications to CYCLOPS for these issues. In this context, noncircadian covariates and batch effects in processing can likely overwhelm circadian variation. As we demonstrate in our benchmarking, batch-correcting approaches like COMBAT that attempt to “normalize away” these batch differences are unlikely to overcome this obstacle. If different centers have different biases in collection time, that approach may remove true circadian signal. This challenge is particularly relevant when combining clinical biopsy and autopsy-based collections. CYCLOPS 2.0 explicitly accommodates these issues, finding batch and covariate adjustments to utilize a common underlying periodic structure for all datasets.

Notably, circadian rhythms in noncancerous breast tissue are likely to have important clinical implications. Such rhythms can modulate local drug toxicity or radiation-induced injury. Our CYCLOPS 2.0 ordering of noncancerous breast tissue is consistent with well-established circadian physiology (i.e., the relative phase relationships between core clock genes are well preserved) and meets various informatic quality checks. While we cannot fully dismiss the influence of noncircadian variability on the ordering process, our hybrid experimental design lends considerable reassurance. We used a small number of newly collected time-stamped samples to guide our efforts and demonstrate that the informatic ordering reflects natural temporal variation.

Among breast cancer subtypes, luminal A tumors had the most robust evidence for persistent rhythms, prompting our interest in using informatic tools to order these tumor tissues along circadian time. We find marked changes in the informatically reconstructed cycling in luminal A tumors; many genes and pathways, including chemotherapeutic targets, gained or lost rhythmicity. Using CYCLOPS magnitude as a measure of the global rhythm strength in each sample, we identify marked variations in the rhythm strength of luminal A tumors. The global magnitude of rhythmic oscillation in luminal A tumors predicts 5-y survival and positively correlates with cycling in EMT pathway genes. Our in vitro experimental evidence casually links molecular clock disturbance with cancer cell invasion in a 3D model, thus supporting our informatic result. The work of De et al., who observed MCF-7 cells and noted circadian rhythms in EMT-associated changes in cell morphology (65), also buttresses our results.

A previous analysis of TCGA data compared mRNA levels of paired tumor and nontumor samples in 14 cancer types. They report a similar downregulation of *PER1*, *PER2*, *CRY2*, and *HLF*, in breast cancer samples while *CLOCK*, *ARNTL*(*BMAL1*), and *BHLHE40* levels remained relatively unchanged (66). However, by including a temporal ordering component in the analysis of luminal A tumors, our hybrid design allowed us to use TCGA data to assess changes in the rhythmic expression of clock genes and clock outputs important to cancer biology. For example, we observe that *ARNTL*(*BMAL1*) loses rhythmicity in addition to a change in basal expression. Similarly, while *TEF* cycling shows reduced amplitude, its functional paralogue (67) *DBP* shows increased cycling amplitude in luminal A tumors. These data will allow cancer researchers to identify chemotherapeutic targets with temporal properties which differ between cancer and noncancerous tissue, opening a potential chronotherapeutic opportunity. For example, we note cycling in *BRAF* and other kinase pathway target genes (Datasets S4–S8). Established breast cancer targets *CDK6* and *EGFR* also show high-amplitude rhythms. Drug target pathway enrichment analysis suggests that targets of Doxorubicin and Cisplatin, two established breast cancer therapeutics, are enriched for genes that cycle in luminal A tumors. Highly rhythmic luminal A tumors, which demonstrate increased invasiveness and a relatively poor prognosis, are likely to be most responsive to time-aware therapies.

Indeed, our data suggest that CMag, or the amplitude of EMT rhythms, has prognostic value beyond the simple expression level. We computed the average expression of all genes assigned to the EMT Hallmark pathway and separately considered the expression of canonical EMT genes *CDH1*, *CDH2*, and *TWIST1*. Dividing the samples into tertiles by CMag, we found no significant differences between the groups in gene expression levels (*SI Appendix*, Fig. S9 *A–D*). Except for *CDH2* (*SI Appendix*, Fig. S9*C*), gene expression SEM increased with the magnitude group, corresponding to increasing gene amplitude by the magnitude group (*SI Appendix*, Fig. S9 *A*, *B*, and *D*). Alternatively, dividing the samples into tertiles based on the expression of the *CDH1*, *CDH2*, or average Hallmark EMT gene expression did not offer prognostic significance (*P* > 0.05 GLM) (*SI Appendix*, Fig. S9 *E–G*). While grouping samples based on *TWIST1* expression did appear to have a trend for prognostic value (*P* = 0.07 GLM) (*SI Appendix*, Fig. S9*H*), this was distinct from the predictive value of CMag. The addition of CMag to a GLM model that included *TWIST1* expression remained significant (*P* < 0.01).

These insights bring us one step closer to personalized circadian medicine. Comparing rhythms in nontumor and luminal A tumor samples, the EMT pathway genes stood out for demonstrating increased cycling in tumor samples. Comparing cycling between luminal A tumors with high/low global rhythm strength again highlights the EMT pathway. Our repeated identification of EMT as a rhythmically coordinated pathway in luminal A tumors is particularly intriguing, given recent reports that the metastatic spread of breast cancer accelerates during sleep (68). Although our experimental results show that luminal A tumors have cell-autonomous rhythms, our informatic study cannot distinguish direct clock outputs from rhythms imparted from cycling hormones or other physiological signals. Even among tumors with cell-autonomous rhythms, some tumors might be more easily entrained by environmental or physiologic signals. Tumors with high rhythm magnitudes may be more responsive to host signals. Sleep, for example, is associated with changes in autonomic tone, body temperature, hormone secretion, and food intake that all have the potential to entrain responsive tumor clocks. Using mouse models, Hill and colleagues previously identified nocturnal light exposure and the corresponding change in host melatonin rhythms as influencing EMT (69). While the association between tumor rhythm strength, EMT cycling, and patient prognosis is important regardless of mechanism, distinguishing these possibilities is likely essential for targeted therapy. Further experiments are needed to evaluate the influences of different zeitgebers (time cues) on entraining tumor rhythms and relative contributions of host and tumor rhythms in modulating disease. The change in in vitro luminal A invasiveness following clock disruption suggests that tumor-autonomous rhythms causally influence metastatic potential. However, it is also possible that circadian fitness or responsiveness is a marker of other features that contribute to an aggressive phenotype.

Of particular note, our result suggesting that tumor rhythm strength is a potential prognostic marker warrants significant follow-up and prospective verification in an independent cohort before any clinical application. Currently, CYCLOPS uses the complete list of ~70 seed genes to compute the magnitude score. A smaller cohort of transcripts could potentially suffice. While circadian magnitude offers prognostic value beyond PAM50 tumor type and *MKI67* transcript expression, we do not have immunostained Ki-67 protein levels in these TCGA samples. However, we predict that our mechanistic insights and the awareness that high-magnitude tumors are better candidates for circadian medicine approaches may prove most useful.

Our results also emphasize the importance of subtype and patient-specific analysis of tumor rhythms. The interactions between cancer biology and circadian rhythms are multifaceted and tumor dependent. Tumors that disrupt *BMAL1* or *CRYs* may lock the clock at different phases, leading to different downstream effects. Notably, there was also a nonsignificant (*P* > 0.05) trend toward poorer outcomes comparing tumors with the weakest rhythms to those in the middle. This trend may reflect just such a dichotomy as in vitro, reduced BMAL1 function led to proliferation. The biological differences between tumor subtypes extend far beyond the clock. Indeed, our screening analysis suggests that more aggressive HER2 and triple-negative tumors have weaker or absent rhythms. Nevertheless, we find that within luminal A tumors, increasing rhythm strength appears to predict increased invasiveness. Our results cannot be applied directly to other tumor types. Future studies could test the intriguing hypothesis that specific cancer cells “hijack” the clock to temporally organize metabolic programs, evade immune surveillance, suppress apoptosis, or facilitate intravasation and metastasis (55). Some cancers may specifically disrupt the circadian check on cell division. Other cancer cells may have broken loose from circadian control altogether (56, 67). For luminal A tumors, tumor rhythms may impart increased biological fitness to the detriment of the patient. Taken as a whole, the biological insights from this study may help lay the groundwork for improved breast cancer prevention (e.g., lifestyle changes), alternative prognostic biomarkers, and more effective personalized breast cancer treatments.

## Materials and Methods

Ethical approval for patient samples: All work involving fresh human tissues was approved by the Human Tissue Authority and the Ethical Approval Committee of the MCRC (Manchester Cancer Research Centre) Biobank Research Tissue Bank Ethics at The Christie NHS Foundation Trust (ref: 18/NW/0092).

Briefly, time-stamped breast tumor and paired normal breast tissues were collected and RNAs isolated for RNAseq studies. Human primary MECs were isolated and cultured as organoids and bioluminescent *BMAL*1-Luc reporter was used to evaluate circadian rhythms in different cancer subtypes. CYCLOPS 2.0 was used to reconstruct rhythms in noncancerous breast tissue and in luminal A cancers from the newly collected samples, as well as from datasets in GTEx and TCGA. The influence of circadian clock rhythm strength on patient prognosis and cancer cell behavior (hanging drop and cell invasion assay) is further determined by CYCLOPS magnitude analysis and by genetic and pharmacological disruption of the molecular clock in cells. The role of ER signaling was assessed by CRISPR-Cas9 KO of *ER* or by chemical modulators of ER. For further details, please see *SI Appendix*, *Materials and Methods*.

## Supplementary Material

Appendix 01 (PDF)

Dataset S01 (CSV)

Dataset S02 (CSV)

Dataset S03 (CSV)

Dataset S04 (CSV)

Dataset S05 (CSV)

Dataset S06 (CSV)

Dataset S07 (CSV)

Dataset S08 (CSV)

Movie S1. **Related to Fig. 2C, Luminal A.** Representative LV200 bioluminescence imaging in human tumor and normal organoids isolated from a Luminal A breast cancer patient.

Movie S2. **Related to Fig. 2C, TNBC.** Representative LV200 bioluminescence imaging in human tumor and normal organoids isolated from a TNBC patient.

Movie S3. **Related to Fig. S8C.** Representative Incucyte real-time live imaging for analysis of cell proliferation of *BMAL1-KD MCF-7* cells.

## Data Availability

All the code and files are available at https://github.com/ranafi/tumor-circadian-clock-strength-influences-metastatic-potential-and-predicts-patient-prognosis.git (70). RNAseq raw data were deposited in the Gene Expression Omnibus (GEO) repository (accession number GSE233242) (71). The authors declare that all data supporting the results in this study are available within the paper and its supporting information.
